# Strategic development of integrated governance for type 2 diabetes Mellitus (T2DM) care in Indonesia: a mixed-methods approach

**DOI:** 10.1080/16549716.2026.2711562

**Published:** 2026-08-06

**Authors:** Dumilah Ayuningtyas, Made Ratna Saraswati, Amaliyah Nurmely Rahmah Saragih, Chiefriana Dearni Gultom, Ruth Christy Setyaningtyas, Hanifah Yazna

**Affiliations:** Department of Administration and Health Policy, Faculty of Public Health, Universitas Indonesia, Depok, Indonesia

**Keywords:** Service quality (SERVQUAL), quality of life (SF-36), multistakeholder coordination, medication adherence, educator

## Abstract

**Background:**

Type 2 Diabetes Mellitus (T2DM) management requires integrated and continuous care; however, service fragmentation remains prevalent in universal health coverage (UHC) systems.

**Objectives:**

This study examines how governance arrangements influence service integration and patient-reported experiences in T2DM care in Indonesia, to develop strategic recommendations for strengthening integrated governance.

**Methods:**

A concurrent mixed-methods design, qualitative driven, was employed. Qualitative data were collected through in-depth interviews with policymakers, insurance representatives, hospital managers, clinicians, and primary care providers Quantitative data were collected from 107 adults with T2DM using structured questionnaires assessing knowledge, medication adherence, perceived service quality, and health-related quality of life. Findings were integrated using a joint display guided by Green’s PRECEDE framework; strategic positioning was assessed through a SWOT and Internal-External matrix, and a TOWS matrix was developed to formulate recommendations.

**Results:**

Qualitative findings identified four governance barriers: fragmented primary–referral care pathways, misaligned financing arrangements, uneven facility and workforce capacity, and weak coordination and monitoring mechanisms. Quantitative findings indicated high patient knowledge (median 86.7%) and medication adherence (76.6% highly adherent), yet generally positive service quality perceptions, though reliability showed the largest gap. Physical quality of life remained suboptimal, particularly in energy and general health. Strategic positioning placed current T2DM governance in the grow-and-build quadrant, indicating capacity for proactive reform.

**Conclusion:**

The development of an integrated governance strategy emphasizing structured referral pathways, aligned financing with chronic care needs, institutionalized diabetes educator roles, and accelerated health information system interoperability – offering actionable lessons for universal health coverage systems in low-and middle-income countries.

## Paper Context

- **Main findings:** Integrated governance of Type 2 Diabetes Mellitus care in Indonesia remains constrained by fragmented referral coordination, limited primary care authority, financing misalignment, and non-interoperable health information systems, despite relatively high patient knowledge, medication adherence, and service quality perceptions.
- **Added knowledge:** This study demonstrates how combining patient-reported outcomes, service quality assessment, and multistakeholder governance analysis within a mixed-methods framework can identify system-level priorities and serve as a basis for developing integrated governance strategies for chronic disease care under universal health coverage.
- **Global health impact for policy and action:** The findings provide practical lessons for low-and middle income-countries on improving chronic disease outcomes through integrated governance, strategic purchasing, strengthened primary care capacity, multidisciplinary coordination, and interoperable digital health systems.

## Background

Type 2 Diabetes Mellitus (T2DM) remains a leading cause of global morbidity and mortality due to chronic complications such as nephropathy, retinopathy, and cardiovascular disease. According to the International Diabetes Federation (IDF), the global adult diabetes prevalence is projected to reach 11.1% in 2024, with over 40% of cases undiagnosed [1]. Indonesia bears a substantial share of this burden, with an estimated 20.4 million people living with diabetes and a national prevalence of 11.3%, driven by aging populations, lifestyle changes, and urbanization [2]. This trend intensifies pressure on the national health system to address both preventive and long-term management needs for chronic diseases like T2DM.

Despite evidence-based international guidelines from the American Diabetes Association (ADA) and the IDF, Indonesia faces significant challenges in their consistent implementation across health facilities [3,4]. Suboptimal outcomes are attributed to limited diagnostic technologies, clinical practice inconsistencies, delayed insulin initiation, and gaps in patient health literacy [5]. Beyond these clinical constraints, evidence increasingly shows that T2DM outcomes are strongly shaped by upstream health system and governance factors. Previous studies demonstrate that diabetes awareness, treatment adherence, and glycemic control are profoundly influenced by health system characteristics, including financing, service delivery models, referral mechanisms, and continuity of care [6]. Fragmentation across care levels, weak primary-referral coordination, and poorly integrated information systems consistently emerge as structural barriers to effective diabetes management, particularly in low- and middle-income settings.

In Asian contexts, care continuity and integration are critical for sustained diabetes control. A systematic review highlights that strategies like coordinated follow-up, multidisciplinary collaboration, and technology-supported monitoring improve glycemic control, medication adherence, and quality of life [7]. However, fragmented service delivery and poor interoperability between providers remain major obstacles to scaling these interventions.

At the individual and provider levels, behavioral and psychosocial factors, such as health literacy, self-efficacy, and patient-provider communication, interact with system conditions to influence self-management [8–10]. This underscores that individual capacity depends on enabling environments such as service access and institutional support. Broader social and structural determinants, such as socioeconomic status and healthcare access, further influence T2DM prevalence and management, with decentralized systems like Indonesia’s amplifying these vulnerabilities through disparities in local implementation capacity and coordination [11].

In Indonesia, national programs including Jaminan Kesehatan Nasional (JKN, the national health insurance scheme) and the Chronic Disease Management Program (Prolanis) – have expanded chronic disease coverage and strengthened primary care delivery. Evidence suggests that structured chronic care programs can improve metabolic outcomes when adequately supported by financing arrangements and referral systems [12]. However, persistent governance gaps including inconsistent referral pathways, limited facility readiness, and weak inter-organisational coordination – continue to undermine the integration of diabetes services, particularly at the primary – referral care interface [13].

Overall, existing evidence suggests that T2DM management reflects the interaction of clinical, behavioral, and health system determinants rather than a purely clinical issue [6,11,14]. While prior studies have examined patient behaviors and primary-care interventions, fewer have explicitly explored how governance arrangements at the referral hospital-level influence service integration and care continuity in Indonesia [7,12,13].

To address this gap, this study adopts a governance-focused lens, to examine how institutional arrangements influence service integration and patient-reported outcomes in T2DM care in Indonesia, employing a mixed-methods approach guided by Green’s PRECEDE framework and supported by strategic analysis tools to develop actionable recommendations for strengthening integrated governance.

## Conceptual framework

This study is guided by an integrated conceptual framework comprising three complementary analytical concepts. The first applies Green and Kreuter’s PRECEDE framework [6,15,16] as the primary analytical lens, examining predisposing, enabling, and reinforcing factors within T2DM care governance to identify structural governance barriers across the diabetes care continuum.

**Figure 1.**
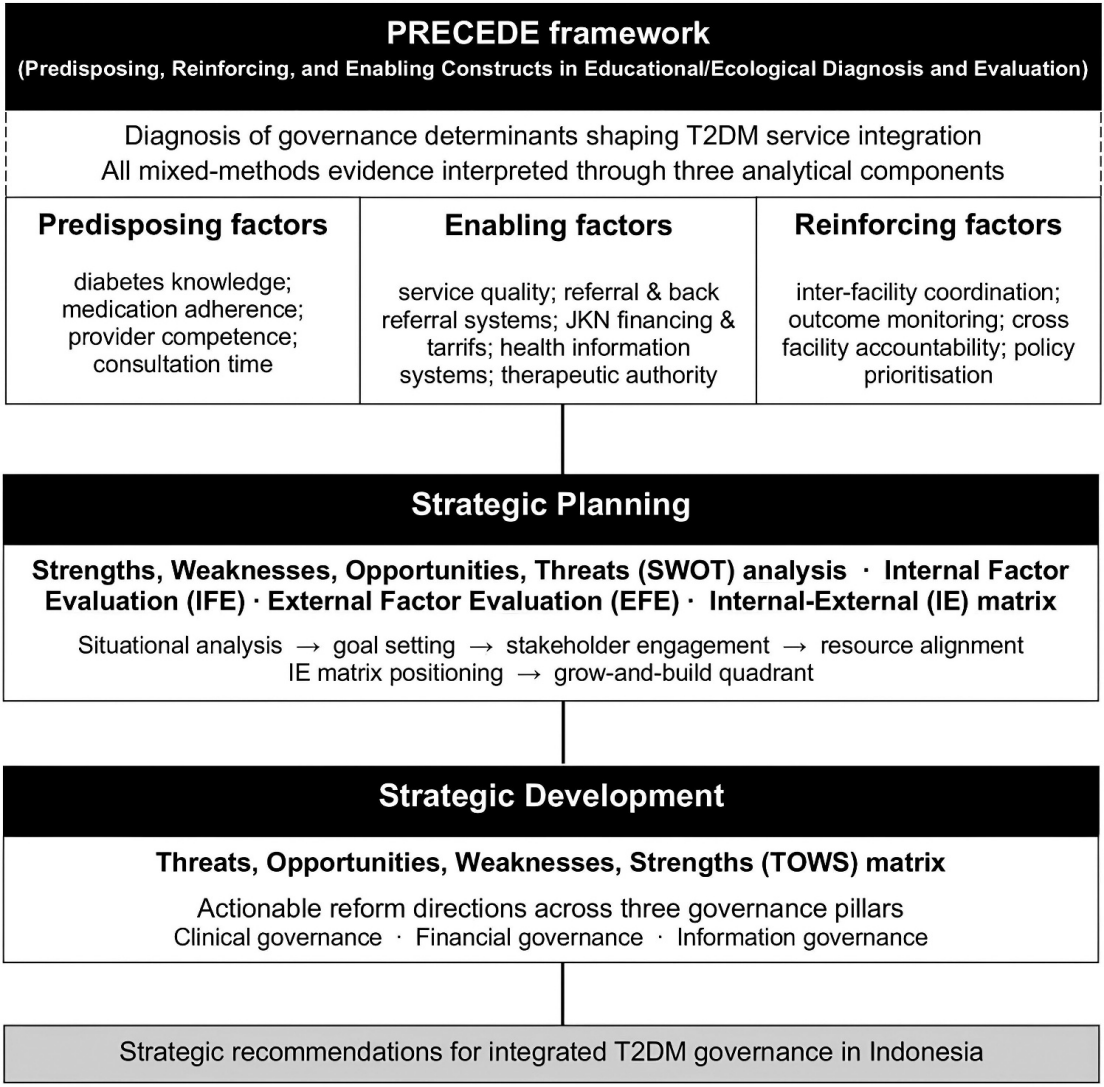
Conceptual framework for the study of integrated T2DM governance in Indonesia.

The second applies a strategic situational analysis using Strengths, Weaknesses, Opportunities, and Threats (SWOT) analysis, Internal Factor Evaluation (IFE) and External Factor Evaluation (EFE) matrices, and the Internal-External (IE) matrix [17], enabling systematic assessment of the internal capacities and external conditions of Indonesia’s current T2DM governance architecture. The third translates the situational analysis into actionable strategic directions using the Threats, Opportunities, Weaknesses, and Strengths (TOWS) matrix [17], which systematically matches internal strengths and weaknesses with external opportunities and threats to generate context-sensitive reform strategies across three governance pillars: clinical, financial, and information governance. The integrated framework is presented in Figure 1.

These three concepts form an integrated analytical framework consistent with a concurrent mixed-methods design [18], in which quantitative descriptive findings provide patient-level evidence that contextualises and strengthens qualitative interpretive analysis. The framework is presented in Figure 1.

## Methods

### Study design and framework

**Figure 2.**
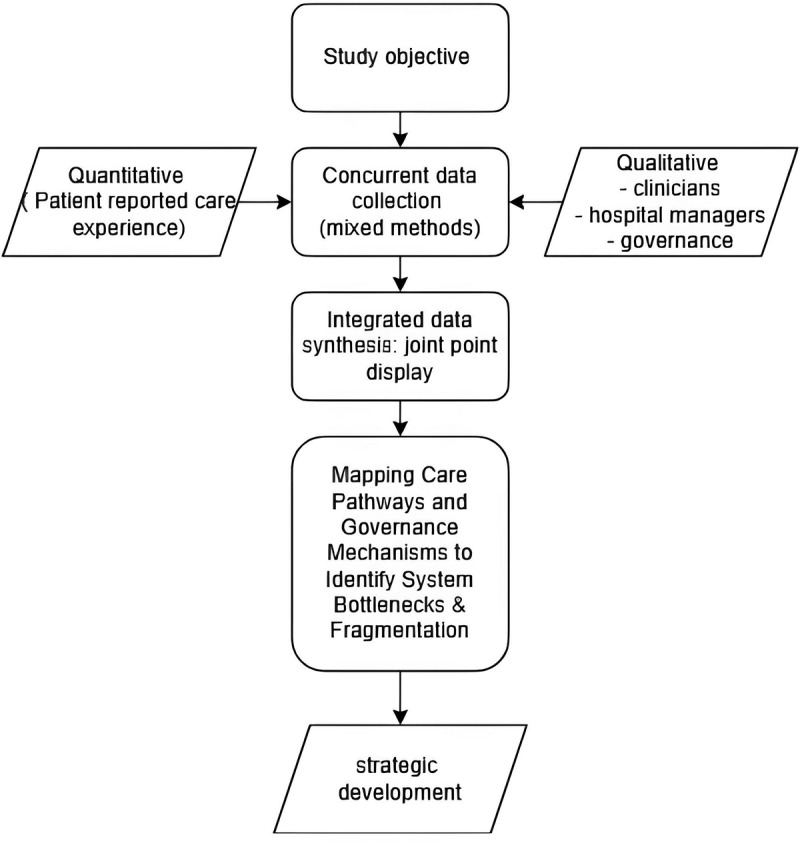
Study framework.

A concurrent mixed-methods design was employed, qualitatively driven, to examine integrated T2DM service governance in Indonesia. The qualitative component constituted the primary source of evidence on governance arrangements, financing mechanisms, and coordination processes, while the quantitative component provided complementary patient-level context on care experiences and self-reported outcomes – not intended to generate nationally representative estimates or test causal relationships. As illustrated in Figure 2, data from both components were collected simultaneously, analyzed separately, and integrated through a joint display mapping patient-reported outcomes alongside qualitative governance themes to identify care pathway fragmentation and governance bottlenecks underpinning the strategic development.

### Data collection

Qualitative and quantitative data were collected concurrently during the same study period, with each component addressing a distinct dimension of integrated T2DM service governance.

Qualitative data were collected through in-depth interviews with key stakeholders, including hospital directors, medical managers, clinicians involved in diabetes care, primary care physicians, non-communicable disease program coordinators, and representatives from BPJS (Badan Penyelenggara Jaminan Sosial) Kesehatan (Indonesia’s Social Health Insurance Administration Agency) and the Ministry of Health. Informants were recruited from 12 institutions across three levels of the health system: national-level institutions, referral hospitals, and primary healthcare facilities (Table 1). Institutions were purposely selected to capture variation in facility type (public and private), level of care (primary care represented by Puskesmas 1, Puskesmas 2, and Puskesmas 3; secondary care by public secondary referral hospitals, type B, and tertiary care by public tertiary referral hospitals, type A, and private referral hospitals, type C), and geographic location (see Table 1).

**Table 1.** Characteristics of qualitative informants.

No	Position	Informant category	Affiliation	Code	Number of informants
**National-level institutions**					
1	Government, as policy maker	Key Informant	Ministry of Health of the Republic of Indonesia - Non-communicable Diseases (NCDs) Program	N1	1
2	Government, as purchaser	Key Informant	BPJS Kesehatan of the Republic of Indonesia (Central Office)	N2	1
3	Government, as purchaser	Key Informant	BPJS Kesehatan of the Republic of Indonesia (Regional Office)	N3	1
**Referral hospitals**					
4	Government, as provider (hospital)	Informant	Public tertiary referral hospital (type A); member of the Indonesian Society of Endocrinology (PERKENI).	H0	1
5	Government, as provider (hospital)	Informant	Public tertiary referral hospital (type A)	H1	4
6	Government, as provider (hospital)	Informant	Public secondary referral hospital (type B)	H2	2
7	Government, as provider (hospital)	Informant	Public secondary referral hospital (type B)	H3	2
8	Business, as provider (hospital)	Informant	Private referral hospital (type C)	H4	1
9	Business, as provider (hospital)	Informant	Private referral hospital (type C)	H5	1
**Primary healthcare facilities**					
10	Government, as provider (primary care)	Informant	Public primary healthcare center (Puskesmas)	P1	3
11	Government, as provider (primary care)	Informant	Public primary healthcare center (Puskesmas)	P2	2
12	Government, as provider (primary care)	Informant	Public primary healthcare center (Puskesmas)	P3	2
**Total of Informants**					**21**

Note: *BPJS Kesehatan = Badan Penyelenggara Jaminan Sosial Kesehatan (Indonesia’s Social Health Insurance Administration Agency); Puskesmas = Pusat Kesehatan Masyarakat (public primary healthcare centre); PERKENI = Perkumpulan Endokrinologi Indonesia (Indonesian Society of Endocrinology). Informant codes: N = national-level stakeholder; H = hospital-level informant; p = primary care informant.*

This set of informants was considered appropriate and adequate for the study’s governance-focused scope: patients’ perceptions of care, captured through SERVQUAL component of the quantitative survey, were treated as a downstream reflection of the governance arrangements described by these institutional informants, rather than requiring direct patient interviews to establish this link. Interviews were conducted either individually or jointly, depending on participant availability, and carried out face-to-face or virtually based on participant preference. A semi-structured interview guide, developed in line with the study objectives and refined through pilot testing, was used to explore governance arrangements, regulatory frameworks, financing mechanisms, and coordination processes influencing T2DM service delivery. All interviews were audio-recorded with informed consent and transcribed verbatim by the research team.

This qualitative component was designed to capture institutional and provider-level governance perspectives along the referral pathway rather than patients’ individual experiences; in-depth interviews with patients themselves were not conducted. Patient-level experience was instead captured through the quantitative component, which assessed patient-reported knowledge, medication adherence, perceived service quality, and quality of life. This division of methods reflects the study’s primary objective of diagnosing governance-level barriers to integration, rather than constructing a care model derived directly from patient narratives. Independent private clinics and practitioners operating outside the BPJS Kesehatan referral pathway were not included in this study and may exhibit distinct governance dynamics not captured here.

Quantitative data were collected using structured questionnaires administered through two approaches: (1) direct completion via Google Forms during outpatient visits, with assistance from clinical staff to ensure accuracy and completeness, and (2) online dissemination to registered T2DM patients who had previously received care at participating facilities across various regions of Indonesia. Eligible participants were adults aged ≥ 18 years with a confirmed diagnosis of T2DM, actively receiving care, enrolled in BPJS Kesehatan, and willing to provide informed consent. Patients with severe mental disorders or terminal illnesses were excluded. All research procedures were conducted in accordance with the principles of the Declaration of Helsinki.

### Data analysis

Qualitative data were analyzed thematically following Braun and Clarke’s six-phase framework [19], involving repeated transcript reading, systematic inductive coding, and collective review to identify, refine, and name themes – with analytical consistency maintained across coders. Thematic saturation was considered achieved when no new codes or governance-related themes emerged from successive interviews across the full set of informants. Emerging themes were subsequently classified into the three analytical components of Green’s PRECEDE framework based on conceptual alignment: predisposing factors (knowledge and capacities of patients and providers), enabling factors (structural and systemic conditions influencing governance and service delivery), and reinforcing factors (institutional mechanisms shaping coordination, monitoring, and accountability). This classification formed the basis for interpreting governance dynamics and their influence on service integration; the mapping of qualitative themes onto these components is presented in Appendix 1.

Quantitative data were analyzed descriptively using frequencies, percentages, medians, interquartile ranges (IQR), means, and standard deviations to summarize patient characteristics, knowledge, medication adherence, service quality, and quality of life. Patient knowledge was assessed using a 15-item instrument adapted from validated diabetes education tools, with scores converted to percentages. Medication adherence was measured using a five-item self-reported questionnaire scored on a five-point Likert scale and transformed into a standardized 0–100 scale, with higher scores indicating better adherence; adherence levels were categorized as poor (<60%), moderate (60–79%), and high (≥80%). Perceived service quality was assessed using the SERVQUAL instrument [20], which evaluates gaps between patient expectations and perceptions across five dimensions using a seven-point Likert scale; gap scores were calculated as perception minus expectation (P−E). Both the adherence scale and SERVQUAL expectation items demonstrated acceptable internal consistency (Cronbach’s alpha > 0.60). Health-related quality of life was measured using the RAND 36-Item Health Survey (SF-36) [21], with domain scores transformed to a standardized 0–100 scale. All analyses were conducted using SPSS version 25 and Microsoft Excel. No inferential statistical analysis was performed; all quantitative findings are presented as descriptive and exploratory, intended to characterise patient-reported experiences of T2DM care and to provide contextual grounding for the qualitative governance analysis, rather than to test hypotheses or establish causal relationships.

Integration of qualitative and quantitative findings was conducted at the interpretation stage, where quantitative results were used to corroborate and contextualize themes emerging from qualitative analysis, particularly in relation to governance, coordination, financing, and policy implementation. A joint display was developed to illustrate how quantitative patient-level findings and qualitative governance themes converged and complemented each other across the analytical framework (see Table 7).

The research team comprised public health and health policy researchers with expertise in health systems governance. This background may have oriented the analysis toward structural and governance explanations over individual or clinical ones. One team member is a practicing clinician specializing in internal medicine, whose insider perspective contributed clinical insight while also potentially foregrounding clinical dimensions of governance. This positionality was addressed through team-based analysis and triangulation across multiple informant groups and data sources.

## Results

### Qualitative findings

#### Characteristics of informants

The qualitative component involved informants from multiple institutions across multiple levels of diabetes care and governance in Indonesia. All informants were anonymized as presented in Table 1; informant codes are used to attribute quotations throughout the qualitative findings.

#### Fragmentation of care and referral pathways

##### Discontinuity between primary care and referral hospitals

Findings revealed substantial discontinuity between primary healthcare facilities (Puskesmas) and referral hospitals. Governance responsibilities are structurally divided, with primary care positioned for general management and hospitals for advanced clinical interventions, resulting in a fragmented care trajectory reliant on administrative mechanisms.

There are two governance domains: primary healthcare facilities (FKTP) and clinical services. Currently, non-communicable disease programs focus on FKTP, while clinical services mainly serve as referral providers to hospitals, after which hospitals manage treatment. (N1)

This division fosters mutual distrust: *‘Primary care wants to be trusted, while referral hospitals demand proof of capacity. It’s truly a chicken-and-egg problem.’ (N3)*

Consequently, follow-up is weak, with hospitals reporting *“Patients scheduled for three-month follow-ups often disappear after back-referral.” (H2)“The biggest problem is follow-up and patient retention … Many patients seek treatment for one to two months and then stop”. (H0)*

##### Administrative nature of referral and back-referral processes

The Back-Referral Program (PRB) functions as the main formal coordination mechanism but remains largely administrative rather than clinically integrated:

Coordination occurs through the Back-Referral Program (PRB), which captures patients referred to hospitals and then returns them to FKTP. (N1)

Primary care providers reported limited capacity to monitor patients during hospital episodes:

How can FKTP monitor patients who have been referred? They cannot be fully monitored once they are in hospital care. (N1)

Incentive structures further influence referral behavior:

Hospitals are reluctant to refer patients back because outpatient visit numbers affect their revenue. (N3)

Primary healthcare facilities are hesitant to manage diabetes patients who are still receiving adjunct medications from hospitals. (N3)

Operational barriers related to PRB medication access also undermine continuity:

Back-referral is good for reducing the burden on referral hospitals. However, the problem lies with PRB medications: PRB pharmacies are often located far … medication stock is frequently unavailable … and patients who should receive a one-month supply are sometimes given only one week and asked to return again. (H0)

##### Limited therapeutic escalation and clinical follow-up at the primary care level

Primary care providers described constrained clinical authority, particularly regarding insulin therapy:

In terms of competence, we are capable, but insulin dosing is locked by the BPJS system and specialists. (P1)

This leads to ineffective visits. *“Patients keep coming back with high blood glucose levels, but our room to maneuver is very limited”. (P1)*

Hospital informants estimated that *“About 60% of cases could be managed by general practitioners if they were properly trained … Good clinical algorithms become ineffective when the necessary medicines are not available”. (H0)*

##### Absence of meaningful information exchange across levels of care

Fragmented information systems prevent comprehensive clinical handover across care levels. Back-referral documentation often lacks essential data:

Back-referral letters usually only include medications; laboratory results are often missing. (P2)

Claim-based systems like V-Claim are designed for reimbursement, not coordination: *“V-Claim only covers medical claims and does not include detailed laboratory results”. (N2)*

Consequently, continuity relies on patients as information carriers, complicating comprehensive care.

It is difficult to trace care from end to end – whether eye examinations have been done, whether foot assessments have been completed. In the end, the focus narrows to medications, while consultations are often delayed or missed. (H0)

#### Financing and purchasing arrangements for chronic diabetes care

##### The role of JKN and BJPS Kesehatan in shaping service design

Financing arrangements strongly shape delivery. T2DM is positioned as a primary-care condition *“Diabetes does not meet the referral criteria according to clinical practice guidelines, so it should remain managed at primary care facilities under capitation-based financing”. (N2)*

Simultaneously, BPJS views T2DM as a strategic financial priority.

Diabetes mellitus is a disease we consider very special … DM and hypertension are the two main conditions we focus on, both in terms of financing and performance targets. (N3)

##### Outpatient tariffs constraints

Hospital informants described misaligned tariffs. *“If we provide services equivalent to a type A hospital, the claim is still reimbursed at type B rates”. (H2)*

Limits on reimbursable consultations fragment care delivery:

There are limitations on consultations and diagnostic tests – no more than three items are allowed. If we want to provide comprehensive, ‘one-stop’ care, it may only be completed after three months, delivered in stages. (H0)

This leads to fragmented, multi-visit care. *‘It becomes episode one, two, three, four … so patients have to keep coming back and forth.’ (H0)*

##### Financing implications for education and multidisciplinary services

Patient education and nutrition counseling are insufficiently incentivized:

Education is important so patients understand their condition, but there is no specific reward for providing education. (H2)

High patient volumes in primary care further restrict time for individualized education. *“More comprehensive education is usually delivered during monthly Prolanis activities”. (P1)*

Educator roles are not institutionalized within remuneration systems:

Educators are a major problem … the term ‘educator’ does not exist in the remuneration system … education work takes a long time, is demanding, but is not counted as performance. (H0)

This fosters a prescription-focused encounter pattern. *‘The pattern becomes: patients come in, are given prescriptions … before the patient even sits down, the prescription is already written.’ (H0)*

In response, hospitals employ sustainability strategies like cross-subsidization and service innovation.

BPJS patients make up around 85 to 87 percent of our monthly visits, so we need cross-subsidies from private patients. (H3)

#### Facility readiness and workforce capacity

##### Variation in facility readiness

A clear variation exists between primary care and hospital readiness. Puskesmas are equipped for screening and basic management.

Screening can be conducted both inside and outside the facility. When random blood glucose is high, patients can directly undergo HbA1c testing at the primary care facility. Based on those results, physicians assess whether a diabetes diagnosis can be established. (P1)

In fact, diagnosis can be fully established at primary care facilities. Referral to hospitals is not always necessary as long as the case can still be managed at the primary care level. (P2)

However, diagnostic accuracy and access to HbA1c testing remain uneven: *‘HbA1c testing is not available in all centers, and not all primary care facilities have access to it either.’ (H0)*

Advanced management requires referral: *‘Our laboratory facilities are not yet complete enough to detect diabetes complications at an early stage. Advanced examinations still have to be referred to secondary or tertiary care facilities’ (P3).*

##### Human resource constraints and limited consultation time

High patient volumes limit consultation time: *‘With around 200 patient visits per day, individual education sessions cannot be conducted at length’ (P1).*

This results in prescription-focused encounters: *‘In Puskesmas, daily visits can reach 150 patients with only three doctors, so there is essentially no time for education. The pattern becomes: patients come, receive medication, and leave.’ (H0)*

#### Coordination, monitoring, and accountability across institutions

Formal coordination mechanisms exist but are insufficient for clinical continuity: *‘Information we receive from hospitals is often clinically incomplete’ (P1).*

Informal channels are used to compensate: *‘We are part of a citywide JKN Telegram group to coordinate with hospitals and other facilities, but responses are often slow and not effective for managing clinical cases’ (P2).*

A major barrier is the absence of a cross-facility diabetes registry: *‘Back-referral exists administratively, but we do not yet have a cross-facility registry to monitor ongoing therapy and patient outcomes.’ (H1)*

This limits outcome accountability: *‘Outcomes like HbA1c become biased because there is no cross-facility registry. Patients who are referred back are not fully monitored.’ (H2)*

National prioritization remains limited: *‘There is no national diabetes registry yet. Barriers include data privacy concerns and the perception that diabetes is not as urgent a priority as other diseases.’ (N1)*

Fragmented data also constrain digital innovation: *‘I am still hesitant about AI (artificial intelligence) because it requires large amounts of data, and the data are often incomplete. Physical examination items are frequently missing, and laboratory tests that should include around 20 parameters often only have 10.’ (H0)*

Teleconsultation is viewed as feasible for stable patients. However, limited interoperability between health information systems – including e-Puskesmas (the primary care electronic medical record platform) and SIMRS (the hospital management information system) – constrains care continuity across facilities, prompting calls for a unified national platform like Satu Sehat (Indonesia’s national health data interoperability initiative).

Primary care uses e-Puskesmas, while hospitals use SIMRS. There is no direct connection, so we cannot access detailed hospital medical records. (P3)

Access to a ‘one patient, one record’ system across facilities, such as Satu Sehat, would greatly support continuity of care. (H1)

##### Leadership and policy prioritization

Leadership perspectives highlighted misalignment in policy prioritization: *‘Diabetes is one of the diseases that consumes the largest share of JKN financing and serves as an important performance indicator for BPJS.’ (N2)*

At the national level, priorities are still heavily focused on heart disease, stroke, and cancer. Diabetes is more often promoted as a local priority. (H1)

This actor-based presentation enables the identification of areas of misalignment and critical bottlenecks in the implementation of integrated diabetes services in Indonesia (Appendix 4).

### Quantitative findings

#### Sociodemographic and clinical characteristics of type 2 diabetes mellitus patients

A total of 107 patients with T2DM were included: predominantly male (57.0%), with a median age of 56 years, mostly secondary educated or above (73.8%), yet socioeconomically vulnerable (82.2% earning ≤ 5 million IDR monthly). Most had long-standing disease (77.6% >1 year) and multimorbidity (83.2%) (Table 2).

**Table 2.** Characteristics of respondents.

Characteristics	n	%
**Sex**		
Male	61	57.0
Female	46	43.0
**Age (years)**		
Median (Min–Max)	56 (19–91)	
**Highest Education Level**		
No formal education	5	4.7
Elementary school	18	16.8
Junior high school	5	4.7
Senior high school	37	34.6
Diploma	4	3.7
Bachelor’s degree	33	30.8
Master’s degree	5	4.7
**Current Employment Status**		
Unemployed	19	17.8
Housewife	21	19.6
Civil servant	5	4.7
Private employee	25	23.4
Informal worker (self-employed/freelance)	17	15.9
Retired	20	18.7
**Monthly Income**		
No income	36	33.6
<3 million IDR	26	24.3
3–5 million IDR	29	27.1
5–10 million IDR	7	6.5
>10 million IDR	9	8.4
**Duration of Diabetes Mellitus**		
Newly diagnosed	4	3.7
<1 year	20	18.7
1–5 years	40	37.4
>5 years	43	40.2
**Comorbidity Status**		
Yes	89	83.2
No	18	16.8

#### Patient knowledge of T2DM

The median knowledge score was high at 86.67% (IQR: 13.33), yet notable gaps persisted around prediabetes: most patients incorrectly equated its cardiovascular risk with normal glycemic status (72.9%) or believed diabetes always presents with severe symptoms (62.4%), while smaller proportions misidentified its diagnostic criteria (29.9%) or definition (25.2%) (Table 3, Appendix 2).

**Table 3.** Diabetes-related knowledge scores and selected knowledge gaps among patients with type 2 diabetes mellitus.

Domain	Indicator	Value
Overall knowledge score	Median (Inter Quartile Range), %	86.67 (13.33)
	Min-Max, %	40.00–100.00
	Skewness	−1,226
Selected knowledge items (highest incorrect responses)	Incorrectly believing cardiovascular risk in prediabetes equals normal status (%)	72.9
	Misconception that diabetes always presents with severe clinical symptoms (%)	62.4
	Failure to identify blood glucose or HbA1c as diagnostic criteria for prediabetes (%)	29.9
	Failure to correctly define prediabetes as an intermediate glycemic state (%)	25.2

Note: Detailed item-level distributions of patient knowledge are provided in Appendix 2.

#### Medication adherence among patients with T2DM

Medication adherence was generally high (median: 86.00%, IQR: 16.00), with 76.6% of patients classified as highly adherent. The most frequently reported non-adherence behavior was forgetting to take medication (15.9%); other behaviors were less common (5.6–6.6% each) (Table 4, Appendix 3).

**Table 4.** Medication adherence scores, levels, and non-adherence behaviors among patients with type 2 diabetes mellitus.

Domain	Indicator	Value
Overall adherence score	Median (IQR), %	86.00 (16.00)
	Min-Max, %	20.00–100.00
	Skewness	−1.624
Adherence level	High adherence, n (%)	82 (76.6)
	Moderate adherence, n (%)	14 (13.1)
	Poor adherence, n (%)	11 (10.3)
Frequently reported non-adherence behaviours (always/often)	Forgetting to take medication (%)	15.9
	Using less medication than prescribed (%)	6.6
	Skipping medication doses (%)	6.6
	Changing medication dose independently (%)	5.6
	Discontinuing medication without consultation (%)	5.6

Note: Detailed item-level distributions of medication adherence behaviors across all Likert response categories are provided in Appendix 3.

#### Patient perceptions and expectations of SERVQUAL

Service quality perceptions fell short of expectations across all dimensions. The largest gap was observed in Reliability (Gap: −0.27), while Assurance showed the smallest (Gap: −0.19) (Table 5).

**Table 5.** Comparison of patient expectations, perceptions, and service quality gaps across SERVQUAL dimensions.

SERVQUAL Dimension	Expectations (Mean)	Perceptions (Mean)	Mean Gap Score (± SD)	Below Expectations n (%)	Meets Expectations n (%)	Exceeds Expectations n (%)
Tangibles	6.38	6.13	−0.25 ± 0.79	34 (31.8)	56 (52.3)	17 (15.9)
Reliability	6.39	6.12	−0.27 ± 0.77	42 (39.3)	52 (48.6)	13 (12.1)
Responsiveness	6.41	6.17	−0.24 ± 0.81	32 (29.9)	60 (56.1)	15 (14.0)
Assurance	6.41	6.21	−0.19 ± 0.84	29 (27.1)	64 (59.8)	14 (13.1)
Empathy	6.38	6.14	−0.24 ± 0.83	33 (30.8)	60 (56.1)	14 (13.1)
**Overall**	6.39	6.15	−0.24 ± 0.77	45 (42.1)	34 (31.8)	28 (26.2)

Note: *SERVQUAL = Service Quality instrument; SD = Standard Deviation.*

**Figure 3.**
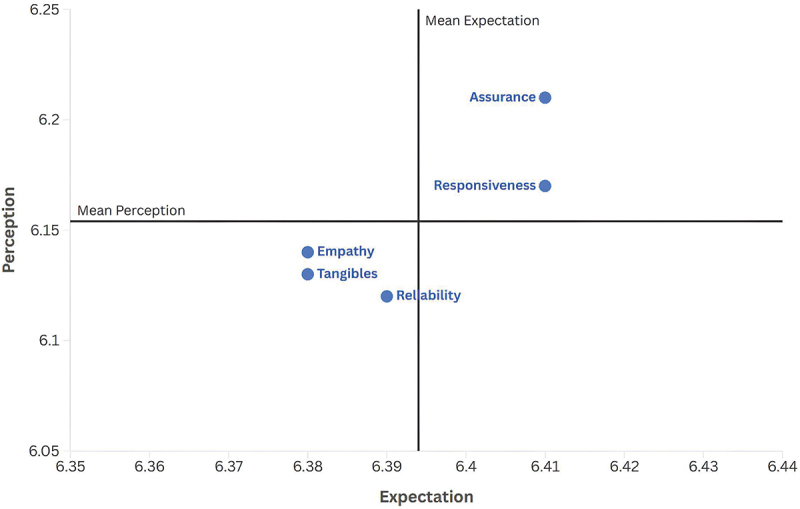
Cartesian mapping of patient expectations and perceptions across SERVQUAL dimensions.

Note: The vertical axis represents mean patient perception scores; the horizontal axis represents mean patient expectation scores. Quadrant boundaries are defined by the overall mean expectation (6.39) and mean perception (6.15). Dimensions positioned in the ‘Keep Up the Good Work’ quadrant indicate relatively higher patient perceptions meeting higher expectations; dimensions in the ‘Low Priority’ quadrant reflect lower expectations with perceptions below the overall mean.

Cartesian mapping positioned Assurance and Responsiveness in the ‘keep up the good work’ quadrant, while Reliability, Tangibles, and Empathy were in the ‘low priority’ quadrant, indicating lower patient expectations for improvement in these dimensions despite the observed gaps (Figure 3).

#### Health-related quality of life (HRQoL)

Physical health (median: 64.6) was more impaired than mental health (median: 70.8), with the lowest scores observed in Energy/Fatigue (58.33), General Health, and Pain (both 62.5) (Table 6).

**Table 6.** Health-related quality of life scores of patients with type 2 diabetes mellitus based on SF-36 domains.

SF-36 Domain	Mean ± SD	Median	Min–Maks
**Summary Components**			
Physical Health Component (PCS)	63.4 **±** 23.9	64.6	14.17–96.9
Mental Health Component (MCS)	66.9 **±** 21.7	70.8	17.08–100.00
**Domain Scores**			
Physical functioning	66.3 **±** 31.1	75	0.00–100.00
Role limitations due to physical health	57.9 **±** 43.7	75	0.00–100.00
Role limitations due to emotional problems	64.8 **±** 43.7	100	0.00–100.00
Energy/fatigue	58.2 **±** 19.4	58.33	8.33–100.00
Emotional well-being	71.1 **±** 17.1	70	25.00–100.00
Social functioning	69.6 **±** 29.7	75	0.00–100.00
Pain	67.8 **±** 27.2	62.5	12.50–100.00
General health	61.7 **±** 16.1	62.5	29.17–91.67
**Overall QoL score**	**65.1 ± 20.7**	**71.25**	**20.63–96.67**

Note: *SF-36 = 36-Item Short Form Health Survey; PCS = Physical Component Summary; MCS = Mental Component Summary; SD = Standard Deviation; QoL = Quality of Life.*

## Joint display of qualitative and quantitative findings

Table 7 presents a joint display integrating quantitative patient-reported findings with qualitative governance themes, organized according to the PRECEDE framework. Quantitative findings characterize the patient experience of T2DM care delivery, while qualitative themes explain the structural and governance conditions that may underlie those experiences – analytically complementary rather than convergent or causal.

**Table 7.** Joint display of descriptive quantitative findings and qualitative governance themes in type 2 diabetes mellitus care in Indonesia.

Domain	Descriptive Quantitative Findings	Qualitative Governance Theme with Illustrative Quotes	Meta-Inference
**Predisposing**			
Patient education and diabetes knowledge	Median knowledge score: 86.67% (IQR: 13.33). Despite overall high scores, item-level gaps persisted: *• 72.9% incorrectly equated cardiovascular risk in prediabetes with normal glycaemic status* *• 62.4% believed diabetes always presents with severe symptoms* *• 29.9% could not identify diagnostic criteria for prediabetes*	Education delivery was constrained by high patient volumes, limited consultation time, and the absence of institutionalised educator roles in remuneration and performance systems. *“The term ‘educator’ does not exist in the remuneration system … education work takes a long time, is demanding, but is not counted as performance.”* **(H0)** *“The pattern becomes: patients come in, are given prescriptions … before the patient even sits down, the prescription is already written.”* **(H0)**	Item-level knowledge gaps, despite high overall scores, are consistent with qualitative descriptions of uneven and time-constrained education delivery. Both strands point to the need to institutionalise structured diabetes education within routine care.
Medication adherence and self-management support	Median adherence score: 86.00% (IQR: 16.00); 76.6% classified as highly adherent. The most frequently reported non-adherence behavior was forgetting medication (15.9% “always/often”).	Informants described weak follow-up mechanisms, patient loss to follow-up after referral, and fragmented responsibility for continuity between primary care and hospitals. *“Patients scheduled for three-month follow-ups often disappear after back-referral.”* **(H2)** *“The biggest problem is follow-up and patient retention … Many patients seek treatment for one to two months and then stop.”* **(H0)**	High adherence at the time of survey does not capture long-term trajectories of patients who disengage after referral. Both strands indicate that self-management support requires continuity mechanisms beyond the individual encounter, though this study cannot establish a causal link between follow-up gaps and adherence outcomes.
**Enabling Factors**			
Service quality and care reliability	Negative SERVQUAL gaps across all dimensions. Largest gap: Reliability (−0.27 ± 0.77); 39.3% rated reliability below expectations — the highest proportion across all dimensions. Assurance showed the smallest gap (−0.19).	Informants described administrative back-referral processes, weak clinical coordination between Puskesmas and hospitals, and incomplete information exchange across care levels. *“Primary care wants to be trusted, while referral hospitals demand proof of capacity. It’s truly a chicken-and-egg problem.”* **(N3)** *“Back-referral letters usually only include medications; laboratory results are often missing.”* **(P2)** *“It is difficult to trace care from end to end … In the end, the focus narrows to medications.”* **(H0)**	The reliability gap — the largest across all SERVQUAL dimensions — aligns with qualitative descriptions of fragmented referral coordination and incomplete information transfer. These findings are complementary: the quantitative data characterise the extent of patient-perceived unreliability; the qualitative data contextualise its structural origins.
Financing and incentives for integrated care	83.2% of respondents reported comorbidities and 82.2% earned ≤ 5 million IDR/month, indicating that the patient population carries high clinical complexity and socioeconomic vulnerability. *Note: These figures provide patient-level context; they do not directly measure financing adequacy*	Informants described outpatient tariff constraints, consultation limits, and the absence of specific reimbursement for education and multidisciplinary services, creating incentive structures that favor episodic over longitudinal care. *“There are limitations on consultations and diagnostic tests — no more than three items are allowed. If we want to provide comprehensive, ‘one-stop’ care, it may only be completed after three months.”* **(H0)** *“Education is important so patients understand their condition, but there is no specific reward for providing education.”* **(H2)** *“BPJS patients make up around 85 to 87% of our monthly visits, so we need cross-subsidies from private patients.”* **(H3)**	Quantitative findings indicate that many respondents had comorbidities and socioeconomic vulnerability, suggesting potentially complex care needs. Qualitative findings describe financing and reimbursement arrangements that may limit the provision of longitudinal and multidisciplinary diabetes care. These findings are complementary at the contextual level and should not be interpreted as evidence of a direct relationship between financing arrangements and patient outcomes.
Digital information exchange and monitoring	*The quantitative component did not include direct measures of health information system interoperability or digital continuity. This domain is therefore primarily qualitatively driven*.	Informants described fragmented platforms across facilities (e-Puskesmas, SIMRS, V-Claim) with no cross-facility access to longitudinal clinical data and no national diabetes registry. *“Primary care uses e-Puskesmas, while hospitals use SIMRS. There is no direct connection, so we cannot access detailed hospital medical records.”* **(P3)** *“Back-referral exists administratively, but we do not yet have a cross-facility registry to monitor ongoing therapy and patient outcomes.”* **(H1)** *“Access to a ‘one patient, one record’ system across facilities, such as Satu Sehat, would greatly support continuity of care.”* **(H1)**	Digital fragmentation may limit continuity, monitoring, and accountability in T2DM care. As this pathway was not directly measured quantitatively, this domain is informed solely by qualitative evidence
**Reinforcing**			
Existing service capacity and provider responsiveness	Assurance (−0.19) and Responsiveness (−0.24) showed the smallest SERVQUAL gaps and were positioned in the “keep up the good work” quadrant, indicating patients perceived individual providers as competent and responsive.	Informants acknowledged existing capacities, including Prolanis activities and provider commitment, but noted these relied on individual effort rather than institutional systems. *“More comprehensive education is usually delivered during monthly Prolanis activities.”* **(P1)** *“About 60% of cases could be managed by general practitioners if they were properly trained … Good clinical algorithms become ineffective when the necessary medicines are not available.”* **(H0)**	Relatively favorable SERVQUAL scores for provider-level dimensions align with qualitative descriptions of individual provider commitment. Both strands indicate that existing capacities are a system asset, but are currently sustained by individual effort rather than institutional structures.

Note: *SERVQUAL = Service Quality instrument; IQR = Interquartile Range; IDR = Indonesian Rupiah; SIMRS = Sistem Informasi Manajemen Rumah Sakit (hospital management information system); e-Puskesmas = electronic primary healthcare information system; V-Claim = BPJS Kesehatan claims verification system; Satu Sehat = Indonesia’s national health data interoperability initiative; Prolanis = Program Pengelolaan Penyakit Kronis (Chronic Disease Management Program); T2DM = Type 2 Diabetes Mellitus.*

## Integration of PRECEDE findings into strategic development

The integrated findings across the PRECEDE domains (predisposing, enabling, and reinforcing factors), as summarized in the joint display (Table 7), were synthesized into strategic factors for the SWOT analysis, forming the basis for internal strengths and weaknesses and external opportunities and threats. These factors were then evaluated using the Internal Factor Evaluation (IFE) and External Factor Evaluation (EFE) matrices (Table 8), with the researcher serving as the human instrument in assigning weights and ratings through informed analytical judgement based on the integrated evidence in Table 7. Weights reflected each factor’s strategic importance, based on the consistency of quantitative and qualitative findings, its influence on the T2DM referral system, and its relevance to strategic decision-making, while ratings followed the standard IFE/EFE framework, reflecting current organizational conditions for internal factors and organizational responses for external factors. Weighted scores were obtained by multiplying weight by rating.

**Table 8.** Integrating of PRECEDE factors with SWOT analysis.

PRECEDE domains	Code	Strategic factors (SWOT analysis)	Weight	Rating	W. Score
**Predisposing**: • Patient education and diabetes knowledge • Medication adherence and self-management support	S1	The availability of trained diabetes educators at primary health care centers supports structured patient education and self-management for Type 2 Diabetes Mellitus (T2DM).	0.14	3	0.42
**Enabling** : • Service quality and self-reliability • Financing and integrated care • Digital information exchange and monitoring	S2	Primary care facilities receive non-capitation financing and Prolanis claims, providing financial support for chronic disease management activities.	0.13	4	0.52
**Reinforcing** : • Existing service capacity and provider responsiveness	S3	The Prolanis program facilitates patient education, routine monitoring, physical activity, and laboratory referrals every six months, strengthening continuity of care.	0.14	4	0.56
	S4	Health promotion and hospital public relations (PKRS) activities are actively implemented through onsite education, digital platforms, and community-based outreach in schools and local communities.	0.12	4	0.48
	S5	Existing provider commitment and responsiveness support patient trust despite broader system limitations.	0.13	4	0.56
	W1	Although updated national clinical guidelines for diabetes management are available, not all general practitioners have participated in guideline dissemination or training.	0.08	2	0.16
	W2	All SERVQUAL dimensions demonstrate negative gaps between patient expectations and perceptions, with the largest gap observed in the reliability dimension, reflecting issues in consistency and timeliness of care.	0.10	2	0.20
	W3	Communication between primary care and secondary/tertiary care remains limited, particularly regarding continuity of T2DM management after referral.	0.09	2	0.18
	W4	Health service documentation is fragmented across multiple information systems (e-Puskesmas, BPJS Primary Claim, ASIK PTM), limiting data integration and clinical decision-making.	0.07	1	0.07
	**TOTAL IFE SCORE**		**1.00**		**3.15**
	O1	BPJS Kesehatan financing is available to cover essential T2DM diagnostic and monitoring services.	0.25	4	1.00
	O2	Specialist mentoring forums and inter-facility coordination platforms provide a foundation for strengthening clinical collaboration, if reoriented toward operational clinical needs.	0.20	3	0.60
	O3	Integration of the VClaim system enables shared access to patient data across primary and referral care levels.	0.15	3	0.45
	O4	National health information system integration initiatives (SATU SEHAT–BPJS bridging) offer opportunities to enable interoperable patient data access across facilities.	0.14	3	0.42
	T1	Limited community access to health information and education may affect public understanding and health behaviors regarding T2DM management.	0.10	2	0.20
	T2	Poorly coordinated services for patients with multimorbidity may accelerate disease progression and complications.	0.09	1	0.09
	T3	The availability of medical devices and consumables does not always align with BPJS reimbursement schemes, potentially constraining service delivery.	0.07	1	0.07
	**TOTAL EFE SCORE**		**1.00**		**2.83**

Note: *S = Strength, W = Weakness, O = Oppurturnity, T = Threat T2DM = Type 2 Diabetes Mellitus; BPJS Kesehatan = Badan Penyelenggara Jaminan Sosial Kesehatan (Indonesia’s Social Health Insurance Administration Agency); JKN = Jaminan Kesehatan Nasional (National Health Insurance); SERVQUAL = Service Quality instrument; Prolanis = Program Pengelolaan Penyakit Kronis (Chronic Disease Management Program); PKRS = Promosi Kesehatan Rumah Sakit (Hospital Health Promotion); e-Puskesmas = electronic primary healthcare information system; ASIK PTM = Aplikasi Sehat IndonesiaKu Non-Communicable Disease; SATU SEHAT = Indonesia’s national health data interoperability platform, W. Score = Weighted Score (Total)*.

Based on the integrated findings presented in the joint display, strategic internal and external factors were identified and synthesized into an integrated SWOT analysis. The resulting factors were subsequently evaluated using the IFE and EFE matrices (as shown by Table 8) to determine the current strategic position of T2DM referral service governance, providing the basis for the subsequent IE and TOWS matrix analysis in the discussion.

## Discussion

Descriptive survey findings revealed a notable paradox: patients reported relatively high diabetes knowledge and medication adherence, yet physical health-related quality of life remained suboptimal – particularly in energy/fatigue and general health – and service quality fell short of expectations across all dimensions. Qualitative findings, in turn, identified fragmentation across referral pathways, financing arrangements, workforce capacity, coordination, and digital health systems. Together, these findings suggest that patients’ experiences of T2DM care may be shaped more by governance and service delivery arrangements than by individual behavior alone. This discussion interprets these findings through Green’s PRECEDE framework, focusing on predisposing, enabling, and reinforcing factors.

## Predisposing factors: capacity of patients and providers across levels of care

Within Green’s PRECEDE framework, predisposing factors extend beyond individual knowledge and attitudes to include the capacities of health workers and organizations to support sustained health behaviors. This study suggests that patient knowledge and medication adherence reflect not only individual attributes but also how education and follow-up are organized across care settings.

Descriptive quantitative results showed relatively high patient knowledge and self-reported medication adherence. However, qualitative evidence revealed that these capacities exist within a system of fragmented education delivery, limited consultation time, and uneven organizational support. At the primary care level, Puskesmas face extremely high patient volumes (>150–200 visits per day), constraining individualized education and counseling – consistent with evidence that adherence and knowledge may be more influenced by the quality of provider – patient interaction than by information exposure alone [22,23].

At the hospital level, financing and tariff structures restrict consultation duration and multidisciplinary engagement, pushing education and counseling toward informal or secondary roles – consistent with evidence that effective patient education may depend on organizational support and protected time [24,25]. Disengagement from follow-up after referral further illustrates that individual motivation alone may be insufficient without systemic continuity. Evidence highlights that shared care pathways and integrated primary–referral arrangements are important for sustained engagement [26]. PRECEDE-based interventions suggest that self-management requires organizational readiness, adequate consultation time, and structured follow-up mechanisms, without which knowledge and adherence are difficult to sustain [15,16,27,28].

In summary, high knowledge and adherence coexist with service fragmentation, indicating that individual competence alone may be insufficient without system-level support, and that strengthening predisposing factors requires governance reforms enabling longitudinal support at the primary–referral interface.

## Enabling factors: fragmented primary–referral governance as a central barrier

Empirical evidence demonstrates that referral systems, purchasing arrangements, and governance structures linking primary care and referral hospitals function as critical enabling or disabling factors for integrated chronic care within universal health coverage systems [29–31]. This study’s qualitative findings reflect these patterns: governance is structurally divided, with diabetes care formally anchored in primary care while referral hospitals provide episodic interventions without longitudinal responsibility. Continuity of care thus relies heavily on administrative referral and back-referral procedures rather than on shared clinical pathways, further reinforced by mutual distrust between Puskesmas and hospitals regarding clinical responsibility and service capacity [32,33].

National evidence corroborates these constraints: although linkage to diabetes care improved after JKN expansion, fewer than 5% of patients met comprehensive care targets [34]. Financing arrangements further entrench this pattern: case-based outpatient tariffs and narrow benefit packages incentivize episodic over comprehensive care [31,35], and hospital informants reported that JKN tariffs inadequately reflect T2DM’s clinical complexity. Similar financing-driven fragmentation has been documented across both high- and middle-income settings, where misaligned payment mechanisms limit the feasibility of integrated or one-stop care models [36,37], with diabetes-related spending concentrated in secondary and tertiary facilities driven by complications and hospitalization [38].

The largest SERVQUAL gap was observed in reliability – a modest but consistent deficit aligning with qualitative accounts of weak referral coordination and incomplete information exchange. While this study cannot establish a causal relationship between governance arrangements and patient-perceived service quality, the convergence of findings is consistent with the interpretation that referral fragmentation may contribute to perceptions of unreliable care.

Governance fragmentation is further compounded by limited interoperability of health information systems. International evidence identifies integrated clinical information systems as a prerequisite for continuity in chronic disease management [39,40], whereas fragmented information platforms in this study restricted access to longitudinal clinical data and weakened outcome monitoring. These findings suggest that strengthening T2DM services requires more aligned governance, purchasing, and digital systems that support continuity and accountability across care levels.

## Reinforcing factors: institutional coordination, monitoring, and accountability

Persistent gaps in integrated T2DM care may be reinforced by institutional arrangements for coordination, monitoring, and accountability. Despite formal policies supporting referral and chronic disease management, fragmented governance between Puskesmas and referral hospitals continues to undermine longitudinal continuity of care, consistent with evidence from other universal health coverage systems [35,41].

Coordination remains predominantly administrative rather than clinically integrated. Referral and back-referral processes function as episodic handovers, with hospital informants reporting that follow-up responsibility is effectively relinquished at back-referral, while Puskesmas providers described limited clinical authority and incomplete information to resume care – patterns also documented across Europe, Africa, and Australasia [26,42,43]. The descriptive SERVQUAL finding of the largest gap in reliability aligns with qualitative evidence of weak monitoring, including the absence of interoperable health information systems and cross-facility diabetes registries, consistent with evidence that non-interoperable records may impede continuity and accountability [44,45].

Accountability arrangements within JKN financing further sustain these gaps by incentivizing episodic case resolution rather than coordinated follow-up [35,46], reflected in expenditure patterns dominated by hospitalization and complication management [38]. Together, these gaps help explain the coexistence of high patient-reported outcomes with service quality deficits. Without institutionalized coordination, interoperable monitoring systems, and accountability for longitudinal outcomes, integrated care may rely disproportionately on individual effort and informal workarounds [34,47,48].

The governance challenges identified in this study are not unique to Indonesia and may offer transferable lessons for other low-and middle-income countries implementing universal health coverage while managing the growing burden of NCDs. Countries with geographically dispersed or archipelagic health systems – such as the Philippines, Papua New Guinea, and Pacific Island nations – face structurally similar barriers to referral integration and information continuity [30,32]. Evidence from Kenya, China, South Africa, and Australia similarly shows that fragmented referral pathways, misaligned financing, and the absence of interoperable information systems are recurring structural obstacles to integrated chronic care [29,30,32,33].

Three cross-cutting lessons emerge from this study. First, expanding insurance coverage without realigning financing incentives toward longitudinal, multidisciplinary care risks entrenching episodic service patterns [35,36]; strategic purchasing reforms, including tariff adjustments and performance incentives for primary care retention, may be a necessary complement. Second, health workforce constraints at the primary care level, including high patient volumes, limited consultation time, and the absence of institutionalized educator roles, may undermine self-management even where coverage and guidelines exist [30,44]; formalizing these roles within national workforce standards is a potentially high-impact reform. Third, fragmented health information systems disproportionately burden patients by transferring coordination responsibilities to individuals rather than institutions, and addressing interoperability standards early in digital health system development may substantially reduce long-term fragmentation costs [39,40].

## Developing strategy

**Figure 4.**
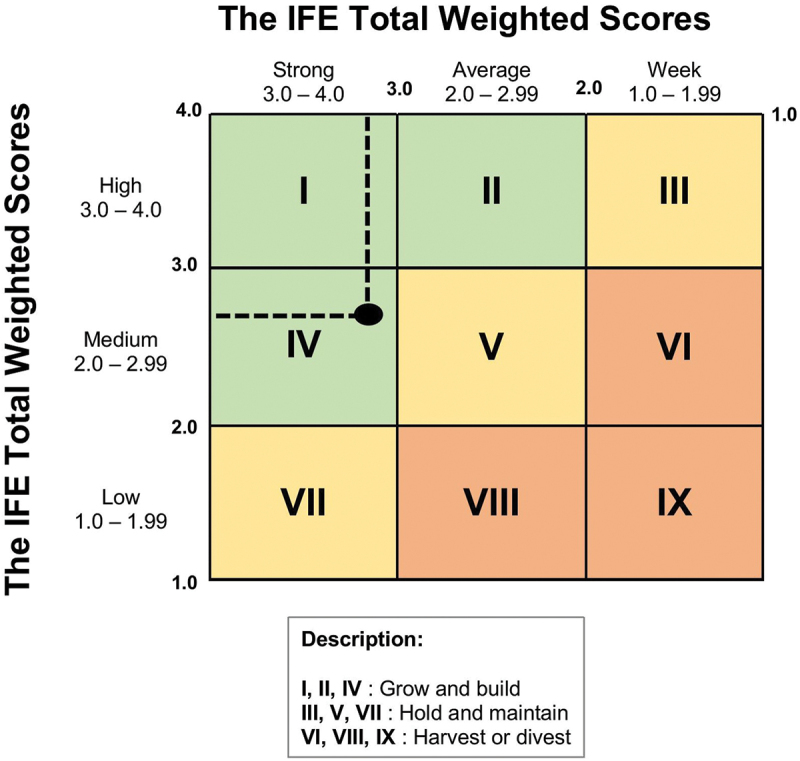
IE matrix.

Table 8 shows that the integrated SWOT analysis resulted in an IFE score of 3.15 and an EFE score of 2.83. These scores place T2DM service governance in the Grow and Build quadrant of the IE Matrix, supporting the development of growth-oriented strategies (Figure 4).

The Grow and Build position identified in the IE Matrix indicates that the current governance of T2DM services is supported by strong internal capabilities and favorable external conditions. Accordingly, the integrated SWOT findings were translated into strategic recommendations through the TOWS matrix by aligning internal and external strategic factors. The resulting strategies are presented in Table 9.

**Table 9.** TOWS matrix.

SO strategies	WO strategies	ST strategies	WT strategies
Strengthening standardized diabetes education models based on Prolanis and trained primary health care educators.	Reorienting specialist mentoring and coordination forums toward operational and clinical problem-solving.	Expanding community-based, continuous education to improve patient adherence and self-management.	Reformulating T2DM service governance based on standardized clinical pathways and integrated referral systems.
Integrating primary and referral clinical services through national digital health system interoperability.	Strengthening PHC clinical workforce capacity through nationally standardized educator training programs.	Optimizing the role of referral hospitals and tiered referral systems to reduce the financial burden of preventable complications.	Adjusting BPJS Kesehatan regulations to support essential medical devices and consumables for T2DM care.
Optimizing non-capitation and Prolanis financing to reinforce promotive and preventive T2DM services.	Integrating T2DM service recording systems across platforms to support clinical decisions and financing efficiency.	Utilizing Prolanis as a quality and cost-containment instrument for T2DM services.	Simplifying and integrating health information systems to enhance service effectiveness and financial sustainability.

Note: *T2DM = Type 2 Diabetes Mellitus; BPJS Kesehatan = Badan Penyelenggara Jaminan Sosial Kesehatan (Indonesia’s Social Health Insurance Administration Agency); PHC = Primary Health Care; Prolanis = Program Pengelolaan Penyakit Kronis (Chronic Disease Management Program).*

While the TOWS matrix outlines strategic options conceptually, effective implementation requires translating these strategies into clearly defined roles and actions across key stakeholders. The proposed strategies were therefore operationalized into stakeholder-specific recommendations involving the Ministry of Health, BPJS Kesehatan, primary health care facilities, referral hospitals, and the community (Table 10). Although the quantitative sample was relatively homogeneous, the convergence of findings across methods supports the robustness of the proposed strategies.

**Table 10.** Actionable high-priority recommendations for strengthening integrated type 2 diabetes mellitus governance in Indonesia.

Priority	Reform Area	Responsible Actors	Implementation Pathways	Feasibility in LMIC Context
1 (Immediate)	Standardize clinical handover across referral and back-referral pathways	Ministry of Health; BPJS Kesehatan; Referral Hospitals; Primary Health Care (Puskesmas)	– Revise PRB documentation requirements via MoH circular to mandate inclusion of laboratory results, active treatment plans, and explicit follow-up schedules in all back-referral letters. – Define minimum clinical data standards for handover between hospitals and Puskesmas. – Monitor compliance through existing BPJS claims audit mechanisms. – Pilot in vertical referral hospitals before national scale-up.	High — builds on existing PRB infrastructure; no new legislation required; operationalizable within the current JKN framework.
2 (Short-to- medium term)	Realign JKN financing to incentivize longitudinal chronic disease management	Ministry of Health; BPJS Kesehatan	– Revise outpatient tariff calculations to reflect multimorbidity complexity and multidisciplinary care needs. – Introduce performance-based incentives for primary care facilities that successfully retain and manage stable Type 2 Diabetes Mellitus patients. – Formalize diabetes educator roles within BPJS remuneration and facility accreditation standards, using existing Prolanis financing model as precedent.	Moderate — requires inter-agency negotiation between MoH and BPJS Kesehatan; Prolanis precedent supports feasibility; phased rollout reduces fiscal risk.
3 (Medium-to-long term)	Accelerate health information system interoperability and establish a cross-facility Type 2 Diabetes Mellitus registry	Ministry of Health; BPJS Kesehatan; Facility operators; Community health workers	– Mandate Satu Sehat platform adoption across all BPJS-contracted facilities and integrate with V-Claim for cross-facility longitudinal patient records. – Develop a national Type 2 Diabetes Mellitus registry to enable outcome monitoring and accountability across care levels. – Expand teleconsultation for stable patients to reduce travel burden and support community-based follow-up. – Engage community health workers and family-based support networks in registry data entry and patient retention activities.	Moderate — Satu Sehat infrastructure partially exists; main barriers are adoption rate and data standardization; international technical assistance available for LMIC digital health integration.

Note: *T2DM = Type 2 Diabetes Mellitus; BPJS Kesehatan = Badan Penyelenggara Jaminan Sosial Kesehatan (Indonesia’s Social Health Insurance Administration Agency); JKN = Jaminan Kesehatan Nasional (National Health Insurance); MoH = Ministry of Health; PRB = Program Rujuk Balik (Back-Referral Program); Puskesmas = Pusat Kesehatan Masyarakat (public primary healthcare centre); Satu Sehat = Indonesia’s national health data interoperability initiative; LMIC = Low- and Middle-Income Country.*

## Limitations

Several limitations should be acknowledged when interpreting the findings. The quantitative component relied on a relatively small, non-probability sampling, which precludes inferential statistical analysis. Patient-reported measures may be subject to recall and social desirability bias; to partially mitigate this, data collection was facilitated by clinical staff and caregivers who assisted participants during outpatient visits. Although deliberate efforts were made to capture geographic diversity across multiple provinces, data collection did not achieve the broader national coverage originally planned; nonetheless, participating facilities spanned a range of institutional capacities – from national tertiary referral hospitals to secondary and primary healthcare centres – capturing meaningful variation in governance arrangements and service delivery contexts. These governance challenges are consistent with broader evidence from other low-and middle-income countries implementing universal health coverage, supporting the relevance of these findings beyond Indonesia.

In addition, the proposed strategic recommendations were informed by stakeholders with governance and decision-making responsibilities, consistent with the study objective of diagnosing governance-level barriers to integrated T2DM service delivery. Consequently, patients’ perspectives were not incorporated directly into the qualitative strategic development process. Nevertheless, patients’ experiences, expectations, and perceptions of healthcare services were systematically captured through the quantitative component and considered alongside the qualitative findings in formulating the proposed strategies.

## Conclusion

Integrated T2DM governance in Indonesia cannot be strengthened through clinical and behavioral interventions alone. The evidence from this study points to a clear imperative: governance reforms must address the structural conditions – fragmented referral pathways, misaligned financing, weak information systems, and diffuse accountability – that prevent individual-level strengths from translating into reliable, continuous care. Operationalizing structured referral-back mechanisms, aligning JKN financing with the demands of longitudinal chronic disease management, institutionalizing diabetes educator roles, and accelerating health information interoperability are not peripheral refinements but foundational prerequisites for integrated care. For Indonesia and other LMICs navigating UHC implementation alongside a rising NCD burden, this study suggests that system-level governance reform is the critical link between coverage expansion and meaningful improvement in patient outcomes.

## Recommendations

The findings suggest that meaningful progress in T2DM care requires reorienting governance from facility-based, episodic delivery toward shared responsibility for longitudinal care, through coherent reforms across clinical pathways, financing, and health information infrastructure. For Indonesia, this reorientation is achievable: existing platforms, including the Back-Referral Program, Prolanis, and Satu Sehat, offer viable foundations for incremental governance strengthening without requiring wholesale system redesign. The remaining challenge lies less in resources than in aligning incentives, information systems, and institutional responsibilities toward continuity and accountability in chronic disease management.

## Supplementary Material

260796558_GRAMMS_and_SRQR_Strategic_Development_of_Integrated_Governance_for_T2DM_Care_in_Indonesia_Dumilah_8_July_2026.docx

260796558_Figure File JPEG_Strategic Development of Integrated Governance for T2DM Care in Indonesia_Dumilah_8 July 2026.zip

260796558_Supplementary File_Strategic Development of Integrated Governance for T2DM Care in Indonesia_Dumilah_8 July 2026.docx

## Data Availability

The data supporting the findings of this study are not publicly available due to ethical considerations, but are available from the corresponding author upon reasonable request.
