# Supplementary material for: Strategic development of integrated governance for type 2 diabetes Mellitus (T2DM) care in Indonesia: a mixed-methods approach
Source: Glob Health Action. 2026 Aug 6;19(1):2711562. doi: 10.1080/16549716.2026.2711562 (PMC13449434; doi:10.1080/16549716.2026.2711562)
Supplement: Supplementary file 1 [file ZGHA_A_2711562_SM9790.docx]

**SUPPLEMENTARY MATERIAL**

**Strategic Development of Integrated Governance for Type 2 Diabetes Mellitus Care in Indonesia: A Mixed-Methods Approach**

**Appendix 1. Mapping of Qualitative Themes onto PRECEDE Framework Components**

| **PRECEDE Component** | **Definition as Applied in this Study** | **Qualitative Themes** | **Key Governance Issues** |
| --- | --- | --- | --- |
| Predisposing factors | Knowledge, competencies, and capacities of providers and organizations that facilitate or hinder integrated diabetes care delivery. | Limited consultation time constraining patient education; restricted therapeutic authority of primary care providers; uneven provider training and guideline dissemination; variation in facility readiness across care levels. | Provider capacity to deliver comprehensive diabetes care is constrained by high patient volumes, limited clinical authority, and uneven access to diagnostic equipment — undermining the enabling environment for self-management support. |
| Enabling factors | Structural and systemic conditions — including governance arrangements, financing mechanisms, and information infrastructure — that make integrated care possible or impossible. | Fragmented primary–referral governance; administrative rather than clinical referral and back-referral processes; misaligned JKN financing incentives; limited therapeutic authority at primary care level; non-interoperable health information systems (e-Puskesmas, SIMRS, V-Claim). | Referral and back-referral processes function as administrative requirements rather than clinical coordination tools; tariff structures and benefit package design incentivize episodic over longitudinal care; fragmented information systems prevent comprehensive clinical handover. |
| Reinforcing factors | Institutional mechanisms that sustain, reward, or undermine desired governance behaviours and accountability arrangements across care levels. | Weak inter-institutional coordination and monitoring; reliance on informal communication channels; absence of cross-facility diabetes registry; limited national policy prioritization of diabetes; prescription-focused encounter patterns driven by financing incentives. | Coordination relies on informal workarounds rather than institutionalized systems; fragmented data infrastructure prevents longitudinal outcome tracking and accountability; financing structures reinforce episodic rather than comprehensive care. |

Note:

*PRECEDE = Predisposing, Reinforcing, and Enabling Constructs in Educational/Ecological Diagnosis and Evaluation; JKN = Jaminan Kesehatan Nasional (National Health Insurance); e-Puskesmas = electronic primary healthcare information system; SIMRS = Sistem Informasi Manajemen Rumah Sakit (hospital management information system); V-Claim = BPJS Kesehatan claims verification system.*

**Appendix 2. Distribution of Correct and Incorrect Responses for Each Knowledge Item among Patients with Type 2 Diabetes Mellitus**

| **No.** | **Knowledge Item** | **Correct**  **n (%)** | **Incorrect**  **n (%)** |
| --- | --- | --- | --- |
| 1 | Overweight and obesity are risk factors for prediabetes and type 2 diabetes mellitus. | 91.6 | 8.4 |
| 2 | Engaging in at least 150 minutes of physical activity per week can help reduce the risk of type 2 diabetes mellitus. | 88.8 | 11.2 |
| 3 | Consuming high-fiber foods, such as fruits and vegetables, can help control blood glucose levels. | 96.3 | 3.7 |
| 4 | Hypertension frequently coexists with type 2 diabetes mellitus and increases the risk of complications. | 87.9 | 12.1 |
| 5 | Having a parent or sibling with diabetes increases the risk of developing type 2 diabetes mellitus by twofold. | 88.8 | 11.2 |
| 6 | Prediabetes is a condition in which blood glucose levels are elevated above normal but do not yet meet the diagnostic criteria for diabetes mellitus. | 74.8 | 25.2 |
| 7 | Fasting plasma glucose testing for diabetes diagnosis is performed after at least 8 hours of fasting. | 83.2 | 16.8 |
| 8 | The diagnosis of prediabetes is determined based on blood glucose levels or HbA1c measurements. | 70.1 | 29.9 |
| 9 | Individuals with prediabetes have the same risk of cardiovascular disease as individuals with normal blood glucose levels. | 27.1 | 72.9 |
| 10 | Individuals with prediabetes can return to normal glycemic status through healthy lifestyle modification. | 85.0 | 15.0 |
| 11 | Diabetes can always be identified by symptoms because it consistently presents with severe clinical manifestations. | 37.6 | 62.4 |
| 12 | The diagnosis of diabetes mellitus is established through blood glucose testing. | 95.3 | 4.7 |
| 13 | Patients with diabetes mellitus are advised to limit the consumption of added sugars and sweet foods or beverages. | 90.7 | 9.3 |
| 14 | In the early stage of type 2 diabetes mellitus, glycemic control can be achieved through diet and physical activity without pharmacological treatment. | 83.2 | 16.8 |
| 15 | Patients with diabetes mellitus are at risk of developing chronic complications, such as kidney, eye, nerve, and cardiovascular disorders. | 95.3 | 4.7 |

**Appendix 3. Distribution of Medication Non-Adherence Behaviours by Frequency among Patients with Type 2 Diabetes Mellitus**

| **No** | **Item** | **Always**  **%** | **Often**  **%** | **Sometimes %** | **Rarely %** | **Never**  **%** |
| --- | --- | --- | --- | --- | --- | --- |
| 1 | Forgetting to take medication | 10.3 | 5.6 | 20.6 | 29.0 | 34.6 |
| 2 | Changing medication dose | 2.8 | 2.8 | 14.0 | 17.8 | 62.6 |
| 3 | Discontinuing medication without consulting a physician | 2.8 | 2.8 | 10.3 | 11.2 | 72.9 |
| 4 | Skipping medication doses | 1.9 | 4.7 | 18.7 | 36.4 | 38.3 |
| 5 | Using less medication than prescribed | 4.7 | 1.9 | 13.1 | 17.8 | 62.6 |

“Always” indicates frequent non-adherence, whereas “Never” indicates optimal adherence.

**Appendix 4. Summary of Actors Perspective on Integrated Governance of T2DM Care in Indonesia**

| **Main Theme** | **Key Issues Identified** | **Ministry of Health Perspective** | **BPJS Kesehatan Perspective** | **Hospital Perspective** | **Primary Care Perspective** |
| --- | --- | --- | --- | --- | --- |
| **Fragmentation of Care and Referral Pathways** | Administrative referrals; weak clinical follow-up; limited information exchange | Referral policies emphasize service eligibility and regulatory compliance, but provide limited operational guidance for clinical continuity across levels of care. | Referral mechanisms are designed to control costs and service utilization, with limited visibility of patients’ clinical trajectories across facilities. | Referral and return-referral processes function largely as administrative requirements, with minimal feedback on patients’ outcomes after referral. | Patients frequently return from hospitals without sufficient clinical information, making follow-up and continuity of care difficult at the primary care level. |
| **Financing and Purchasing Arrangements for Chronic Diabetes Care** | JKN benefit design; outpatient tariff constraints; limited coverage for education and multidisciplinary services | National benefit packages focus on access to essential services, while comprehensive chronic care components remain unevenly institutionalized. | Purchasing arrangements prioritize episodic outpatient encounters, constraining incentives for education, nutrition counseling, and multidisciplinary diabetes care. | Fixed outpatient tariffs limit flexibility to provide comprehensive chronic care services, prompting reliance on internal cross-subsidization strategies. | Financing structures restrict the scope of services that can be delivered at primary care, particularly for laboratory monitoring and structured patient education. |
| **Facility Readiness and Workforce Capacity Across Levels of Care** | Uneven readiness; limited consultation time; shortage of educators and nutritionists; reliance on individuals | Facility and workforce standards are set nationally, but implementation capacity varies across regions and facilities. | Workforce distribution and service readiness are indirectly shaped through contracting and payment mechanisms rather than direct capacity planning. | Specialist availability and diagnostic capacity are relatively stronger, but service integration often depends on individual providers rather than institutional systems. | High patient loads and limited staffing constrain consultation time, leading to a focus on medication provision over comprehensive education. |
| **Coordination, Monitoring, and Accountability Across Institutions** | Weak coordination; absence of cross-facility patient registry; fragmented information systems; limited prioritization of diabetes | Monitoring relies on fragmented information systems, and the absence of a national diabetes registry limits system-wide accountability. | Claims and utilization monitoring are conducted through separate information systems, with limited integration for clinical monitoring and continuity of care. | Lack of interoperable systems prevents hospitals from tracking long-term outcomes of patients referred back to primary care. | Fragmented information systems limit access to patients’ prior clinical data, hindering continuity and outcome monitoring. |

Note:

*T2DM = Type 2 Diabetes Mellitus; BPJS Kesehatan = Badan Penyelenggara Jaminan Sosial Kesehatan (Indonesia's Social Health Insurance Administration Agency); JKN = Jaminan Kesehatan Nasional (National Health Insurance).*
