# Supplementary material for: Strategic development of integrated governance for type 2 diabetes Mellitus (T2DM) care in Indonesia: a mixed-methods approach
Source: Glob Health Action. 2026 Aug 6;19(1):2711562. doi: 10.1080/16549716.2026.2711562 (PMC13449434; doi:10.1080/16549716.2026.2711562)
Supplement: Supplementary file 2 [file ZGHA_A_2711562_SM9792.docx]

GRAMMS

Title: Strategic Development of Integrated Governance for Type 2 Diabetes Mellitus (T2DM) Care in Indonesia: A Mixed-Methods Approach

| **No** | **GRAMMS Item** | **Response** |
| --- | --- | --- |
| 1 | Describe the justification for using a mixed methods approach | As detailed in lines 37-47 pages 4. |
| 2 | Describe the design in terms of priority, sequence, and integration | As detailed in lines 37-47 pages 4, as illustrated in Figure 2 on pages 5. |
| 3 | Describe each method in terms of sampling, data collection, and analysis | Data collection and sampling are described in detail on pages 5 (lines 27–52) and pages 6 (lines 9–33), while data analysis procedures are presented on pages 6 (lines 37–50) and pages 7 (lines 8–30). |
| 4 | Describe where integration occurred | As illustrated in Figure 1 on pages 4. Also, detailed from lines 31-37 on pages 7, lines 40-47 on pages 18, page 19-21 (Table 7), and pages 21 (lines 45-52), pages 22 (lines 8-52) and Table 8. |
| 5 | Describe limitations of this approach | As detailed in lines 14-37 pages 30. |
| 6 | Describe insights gained from integration | As detailed in the Paper Context section in lines 14-30 pages 32. |

SRQR Checklist

Title: Strategic Development of Integrated Governance for Type 2 Diabetes Mellitus (T2DM) Care in Indonesia: A Mixed-Methods Approach

| **Item** | **Topic** | **Response** |
| --- | --- | --- |
| 1 | Title | Identifies the study as qualitative/mixed-methods research and reflects the focus on governance and service integration. |
| 2 | Abstract | Structured abstract summarizing background, objectives, methods, results, and conclusions. |
| 3 | Problem formulation | Fragmentation in T2DM service delivery within UHC systems and limited evidence on governance arrangements in Indonesia. |
| 4 | Purpose/research question | To explore how governance arrangements influence service integration and patient experience in T2DM care. |
| 5 | Qualitative approach and research paradigm | Qualitative descriptive approach informed by health systems and governance frameworks. |
| 6 | Researcher characteristics and reflexivity | Researchers had backgrounds in public health and health policy and engaged in reflexive discussion during analysis. |
| 7 | Context | Study conducted in Indonesian primary and referral healthcare settings within the national health insurance system. |
| 8 | Sampling strategy | Purposive sampling of policymakers, hospital managers, clinicians, and primary care providers involved in T2DM care. |
| 9 | Ethical issues pertaining to human subjects | Ethical approval obtained; informed consent secured from all participants. |
| 10 | Data collection methods | In-depth interviews. |
| 11 | Data collection instruments and technologies | Interview guides; audio recording with consent; field notes. |
| 12 | Units of study | Policymakers, hospital managers, clinicians, and primary care providers. |
| 13 | Data processing | Audio recordings were transcribed verbatim and anonymized prior to analysis. |
| 14 | Data analysis | Thematic analysis using iterative coding and constant comparison. |
| 15 | Techniques to enhance trustworthiness | Triangulation across data sources and methods; team-based coding and discussion. |
| 16 | Synthesis and interpretation | Findings integrated with quantitative results using PRECEDE and SWOT frameworks. |
| 17 | Links to empirical data | Use of illustrative quotations to support analytical themes. |
| 18 | Integration with prior work, implications, transferability, and contribution | Findings extend evidence on governance fragmentation and inform integrated care strategies in LMIC health systems. |
| 19 | Limitations | Qualitative findings reflect context-specific perspectives and may not be generalizable. |
| 20 | Conflicts of interest | No conflicts of interest declared. |
| 21 | Funding | Funding source reported in manuscript. |
